# Smart implanted access port catheter for therapy intervention with pH and lactate biosensors

**DOI:** 10.1016/j.mtbio.2022.100298

**Published:** 2022-05-18

**Authors:** Bruno Gil, Benny Lo, Guang-Zhong Yang, Salzitsa Anastasova

**Affiliations:** a The Hamlyn Centre, Imperial College London, South Kensington Campus, London, SW7 2AZ, UK; b Institute of Medical Robotics, Shanghai Jiao Tong University, Shanghai, 200240, China

**Keywords:** pH sensor, Lactate sensor, Batteryless device, Near field communication, Central venous catheter, Totally implanted access port

## Abstract

Totally implanted access ports (TIAP) are widely used with oncology patients requiring long term central venous access for the delivery of chemotherapeutic agents, infusions, transfusions, blood sample collection and parenteral nutrition. Such devices offer a significant improvement to the quality of life for patients and reduced complication rates, particularly infection, in contrast to the classical central venous catheters. Nevertheless, infections do occur, with biofilm formation bringing difficulties to the treatment of infection-related complications that can ultimately lead to the explantation of the device. A smart TIAP device that is sensor-enabled to detect infection prior to extensive biofilm formation would reduce the cases for potential device explantation, whereas biomarkers detection within body fluids such as pH or lactate would provide vital information regarding metabolic processes occurring inside the body. In this paper, we propose a novel batteryless and wireless device suitable for the interrogation of such markers in an embodiment model of an TIAP, with miniature biochemical sensing needles. Device readings can be carried out by a smartphone equipped with Near Field Communication (NFC) interface at relative short distances off-body, while providing radiofrequency energy harvesting capability to the TIAP, useful for assessing patient's health and potential port infection on demand.

## Graphical abstract

**Figure:**
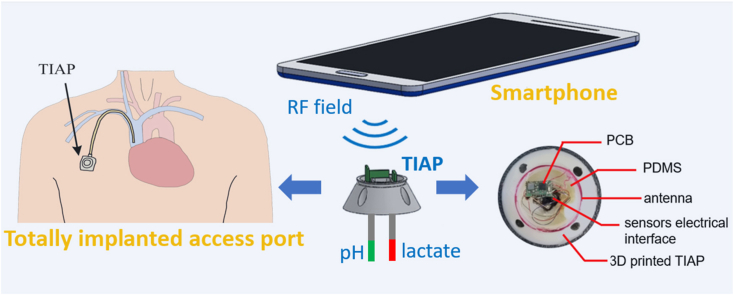

## 1 Introduction

**Fig. 1:**
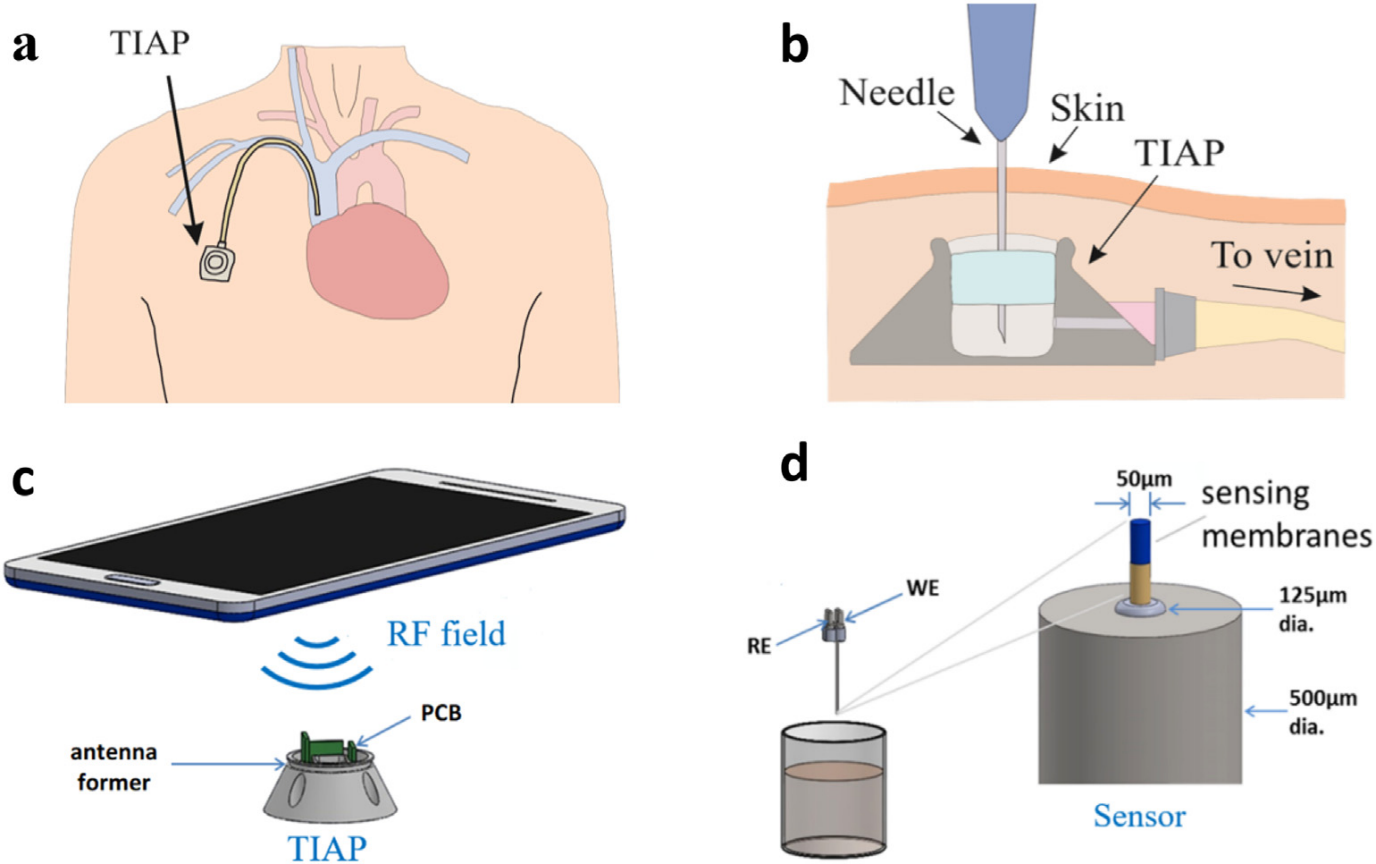
Illustration of: (a) the implantable port catheter device located in the chest for long-term venous access; (b) procedure for port access through skin puncture by a needle; (c) the wireless interrogation scheme for the TIAP; (d) functionalized sensor electrodes (needle) for pH and lactate wireless measurements. Working (WE) and reference (RE) electrodes are developed together with the readout electronics (PCB) and TIAP embodiment.

Central venous catheters (CVC) are widely used with oncology patients that require long-term central venous access for the administration of intravenous medication, blood transfusions and infusions, as well as blood sample collection and parenteral nutritional therapy [1,2]. These devices are therefore essential since chemotherapeutic drugs restrict access to peripheral veins due to cumulative structural damages inflicted to these vessels over time. Apart from oncology applications (solid tumour cancers or haematological malignancies), such devices may also be necessary for patients suffering from human immunodeficiency virus (HIV), cystic fibrosis, and digestive diseases. CVCs can be either external catheters (e.g., the Hickman or Groshong lines) or implanted subcutaneous ports, often referred to as totally implanted access ports (TIAP). This latter solution has the advantage of not restricting patients’ activities in their daily routine, while being associated with significantly lower medical complication rates (e.g., 60% for Hickman lines as compared to 18% with TIAP) and cost treatment [3]. In a typical TIAP, access is provided through a subcutaneous valve located on the chest wall; this in turn connects to a CVC typically inserted into the internal jugular, subclavian or cephalic vein as shown in Fig. 1a. Needle puncture through the skin and silicone membrane covering the port chamber can be required for external access as exhibited in Fig. 1b, though sample collection inside the TIAP for data analysis off-body can be largely replaced by modern contactless sensing mechanisms, drastically improving the well-being of the patient and reducing the risks for transcutaneous tissue infection.

Electrochemical sensors have been deployed throughout the years in several applications including medical diagnostics, environmental monitoring, and manufacturing quality control [[4], [5], [6]]. For example, pH sensors have been used as indicators for wound infection monitoring as the pH level for healthy skin is slightly acidic (between pH 5 and pH 5.5) when compared with infected wounds (pH 7 and pH 8.5) due to the presence of different enzymes and bacteria in the physiological medium [[7], [8], [9], [10]]. Sensing of pH has also been achieved for tissue ischemia monitoring in studies involving excised rabbit and human heart tissue [11], as well as selective tumour detection for point-of-care diagnostics [12]. By its turn, lactate is a vital biomarker for tissue oxygenation level detection and tissue viability assessment, therefore highly relevant in health monitoring applications for athletes performing demanding physical exercises, whereas elevated lactic acid accumulation in the physiological medium for other individuals is normally a marker of infection [4,13]. Typical lactate levels measured in blood for resting individuals vary between 0.5 ​mM and 2 ​mM, while increased lactate levels are observed in cases of deprived oxygen supply occurring during extreme physical exercise whenever the anaerobic threshold level is reached, as well as in patients suffering from diabetes or haemorrhagic shock following tissue injury [14,15].

Lactate increase leads to pH level decline, a condition normally termed as lactic acidosis. Consequently, these analytes can work in symbiose to provide a means for ensuring reliable diagnosis for the medical condition under analysis (that is, multi-modal approach) by reducing the occurrence of individual false positive and false negative outcomes. Lactate is also a major indicator in the prognosis and severity assessment of established sepsis in tissues, multiple organ failure and evaluation of the effectiveness for the applied therapy thereafter. Sepsis arises when the response of the immune system to infection is compromised such that it starts to cause injury to body tissues and organs, with early sepsis onset still being difficult to detect in these days. Sepsis tarnishes lactate clearance, increasing lactate levels in circulation, even when vital signs (e.g., blood pressure, pulse rate, urine output) are apparently normal. It has been also reported that patients suffering from insufficient 24-h lactate clearance (defined by a threshold of 2.5 ​mmol/l) present higher risk for infection development, whereas, as a means of stratifying patients for aggressive infection treatment, some studies suggests that lactate levels equal or greater than 4 ​mmol/l substantially increase the risk for acute-phase death [16,17]. In trauma patients, there is a correlation between lactic acidosis and development of septic shock (and subsequent death) supported by venous lactate measurements in patients [18]. The use of this type of sensing modality is therefore important in order to take informed decisions at earlier stages by clinicians and help identify patients in need of antibiotic therapy or even requiring surgical intervention for control of the infectious tissue foci.

In terms of the measurement mechanism involved, pH is the analyte detected by means of a voltammetry transduction mechanism, with the concentration of protons in the measurement medium increasing the electric potential around an active or working electrode in reference to the solution level. The typically high impedance of this electrode must be matched in the acquisition electronics by a low input-bias current amplifier mounted in a non-inverting topology to reduce the measurement error introduced by the multiplication of the bias current by the electrode's impedance, masking the potential generated by the protons in solution. Lactate uses an amperometry transduction mechanism with immobilization of the lactate enzyme inside a sensitive membrane composing the sensor. Lactate is then ionized, with the resulting ions flowing in accordance to the electric field imposed between the reference and working electrodes, being detected by the latter which transforms the ionic flow into electric current, further amplified and converted to a voltage equivalent by a transimpedance amplifier [4].

Nowadays most electrochemical sensors need to work in tandem with readout electronic circuits that convert the time-varying signals into digitized data equivalents, which convey information about the concentration of target biomarkers inside the body more easily while allowing data interpretation by modern technology. As such, a plethora of biochemical sensing platforms with wireless connectivity have been reported recently in many literature studies [[19], [20], [21], [22], [23]]. The wide infiltration of mobile communication devices to the global population provides an immense opportunity for the research community to develop the next generation of devices with multi-parametric electrochemical sensing capabilities applied to the industrial, medical, and military fields [24]. Moreover, these technological advances in combination with cloud computing approaches, big data analysis and novel computational frameworks pave the way for intelligent sensing, with improved competences, security, and functionalities for a wide range of medical applications [25,26]. This in turn results in promising deployment opportunities in areas such as point-of-care diagnosis and homecare. Portable solutions for wireless biosensing can be broadly divided into battery-operated or batteryless devices [5]. Whilst the formers typically achieve much higher data logging rates, they tend to be bulkier than their batteryless counterparts. The requirement of battery recharge renders clinical applications of such devices difficult for the longer-term since periodic explantation of the device from the body or battery replacement are often required. In addition, batteries are large in dimensions and weight, their chemical constituents are not biocompatible and often corrosive to biological tissues, requiring specialized packaging and preventive measures to avoid any issues related to battery failure and electrical current leakage into tissues. Therefore, the design and use of batteryless devices with embedded biosensors or other signal transduction mechanisms offer an attractive alternative [27,28]. Past examples of near-field based biosensing devices are still relatively bulky and/or possess limited channels to interface electrochemical sensors that hinder their deployment inside the body [[29], [30], [31]]. Moreover, other strategies employing implanted ultrasonic devices are even more resources-depleted due to the reduced amount of energy that can be converted by implanted transducers from the external incident acoustic waves [32,33].

Within this paper, we report the design of an integrated near field communication (NFC) device with acquisition channels for electrochemical sensors that occupies an overall area of 3.75 ​mm ​× ​6.5 ​mm, thus facilitating its deployment inside a 3D printed model of an TIAP, as shown in Fig. 1c. The device is then combined with novel needle-based pH and lactate biosensors to provide a means for clinicians to monitor metabolic processes within TIAP-implanted patients (Fig. 1d), as well as detecting potential complications derived from infection and intervene in a timely manner based on informed decisions provided by real-time analysis of these biomarkers in circulation through the bloodstream. The proposed solution can additionally reduce the burden to patients as it no longer requires skin puncture to retrieve blood samples for biomarkers analysis, while it can be seamlessly adapted to other existing TIAP structures without dramatic alteration of the standard clinical practices.

## 2 Material and methods

### 2.1 Fabrication of lactate and pH biosensors

Lactate oxidase (LOx) from *Aerococcus viridians* was purchased from Sekisui Diagnostics (UK). Polyurethane (Textrin 985) was obtained from Bayer AG (Germany). Bovine serum albumin, phosphate buffer saline tablets (PBS, pH 7.4), sulfuric acid (95%), glutaraldehyde (25% v/v aqueous solution) and other standard reagents were obtained from Sigma-Aldrich (UK). All solutions were prepared in deionized water. Dimethyl sulfoxide (DMSO) and tetrahydrofuran were obtained from BDH (UK). Sulfonated polyester ether sulfone polyether (SPEES-PES) copolymer was a kind gift from ICL Colloids and Polymer Group (UK). UV superglue (Loctite 3211) and silver epoxy were purchased from RS Components (UK). Stainless-steel hollow tubes (inner diameter: 0.125 ​mm) were bought from Goodfellow (UK) and polishing films (aluminium oxide: 1, 3 and 5 ​mm) from Thorlabs (UK). Lactate assay kit from Abnova (KA0833, Taiwan) and pH meter from Thermo Fisher Scientific (Eutech 450, USA) were used to measure the lactate and pH levels of the prepared solutions, respectively.

Combined needle electrodes were used in this study as depicted in Fig. 1d. For the development of the lactate and pH biosensors, platinum, and platinum/iridium (90%:10%) wires (Advent Research Materials, UK) insulated with polytetrafluoroethylene (PTFE, 50 ​μm diameter) were used, respectively for the working electrodes (WE). For the reference electrode (RE), a 50 ​μm-thick polyester insulated silver wire was used. Both electrodes were threaded with heat shrinkable tubing and placed inside the hollow channel of the needle body (diameter of 125 ​μm). In order to expose the metal, the insulation layer was removed from both ends of the wire using a small flame and the exposed portion at the distal ends was connected to a header adapter (electrical interface) with conductive silver epoxy. The proximal ends were polished with alumina slurries (1, 0.3 and 0.05 ​mm). Ag/AgCl reference electrode was prepared using a potassium dichromate reference solution (BASi, US). Cyclic voltammetry was used to assess the working electrode surface. Amperometry measurements were performed in two-electrode mode, using the hollow stainless-steel tube as both the counter and reference electrode and the insulated platinum wire insert as the working electrode. For validation during the intermediary steps of sensor fabrication, an Ivium potentiostat was used and controlled with electrochemistry software (Ivium Soft). Cyclic voltammetry, including electrode cleaning and electro polymerisation, was performed in three-electrode mode using a commercial Ag/AgCl reference electrode and a platinum counter in N_2_ purged solution. Prior to the deposition of coatings, the platinum WE surface was cleaned electrochemically in 50 ​mM sulfuric acid by sweeping potentials between 0.4 and ​+ ​1.5 ​V vs. Ag/AgCl at 100 ​mV/s. For SPEES/PES coating, electrodes were exposed to 10% w/v DMSO solution and left overnight in a vacuum oven at 40 ​°C. The enzyme was immobilized by dip coating in enzyme-glutaraldehyde mixture comprising 60 U enzyme and 20 ​mg BSA at a 2:1 ratio, with 1% v/v glutaraldehyde solution. The mixture was cured for 10 ​min, rinsed in PBS, and dried at room temperature for 2 ​h. Following enzyme immobilization, the lactate biosensor was also coated with a polyurethane film to extend their dynamic range in order to include the potentially higher lactate levels verified in tissues during extensive physical exercise and tissue infection/sepsis, as described previously.

pH sensors were developed through electrochemical anodic electrodeposition from IrOx solution [34]. Depending on the deposition conditions, IrOx is classified as anhydrous or hydrous and it has been proved that hydrous IrOx leads to higher sensitivities [11]. So, electrodeposition from aqueous solution was used for the formation of a pH sensitive membrane. The deposition was achieved using constant current with a three-electrode setup. The voltage between the working and the counter electrodes was set to 0.75 ​V for 45 ​min. *In vitro* experiments demonstrated a super-Nernstian sensitivity with excellent uniformity as shown in the *Results and discussion* section.

### 2.2 Electronic readout circuit

**Fig. 2:**
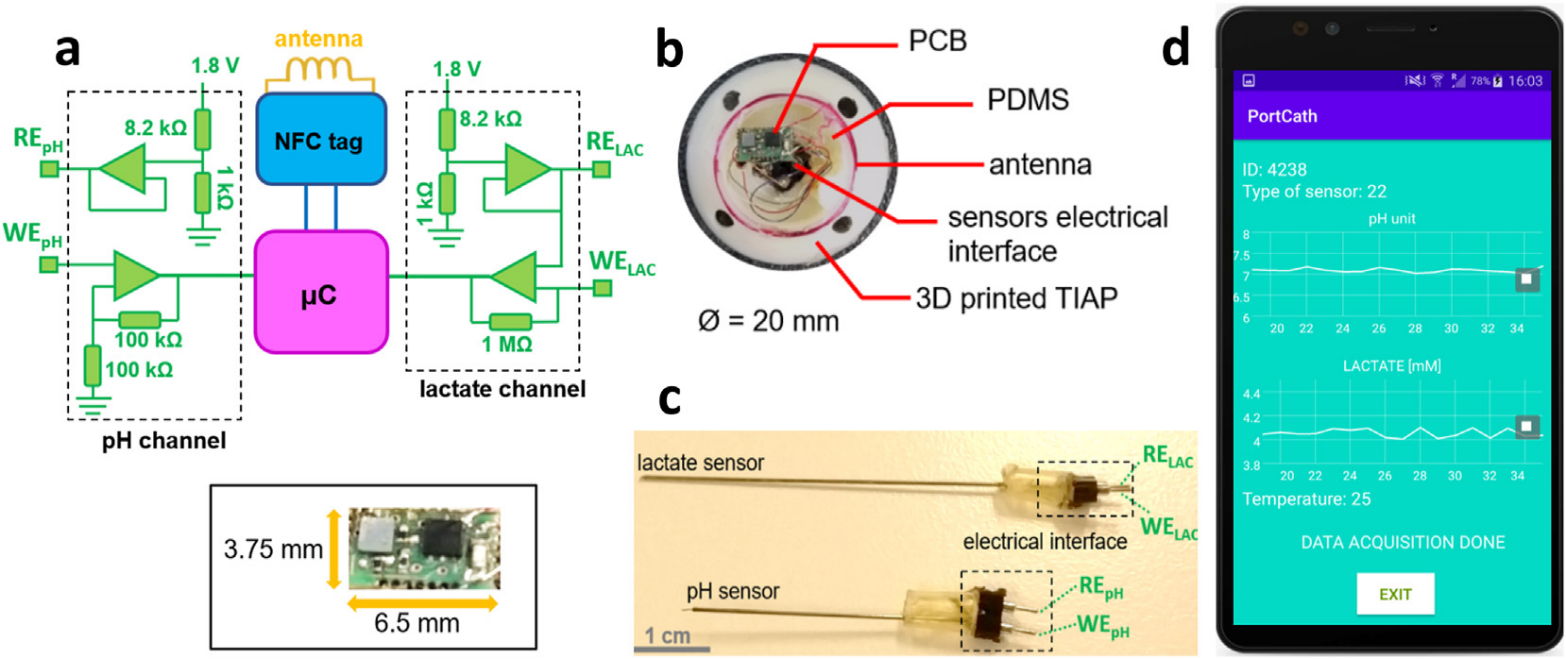
Illustration of: (a) the electronic circuitry composing the device (top) and physical dimension of the printed circuit board (bottom inset); (b) assembly of the smart TIAP device and composing materials; (c) chemical sensors for pH and lactate detection, with measurement tips and electrical interfaces on opposing sides of the needle-like structure; (d) smartphone's *app* developed to receive and visualize the data streams sent from the TIAP device.

The electronics involved in pH sensor readings employed an ultra-low input-bias current amplifier (AD8506, Analog Devices, USA) mounted in a non-inverting topology (gain of 2 ​V/V) and connected electrically to the working electrode for pH (WE_pH_), as shown in Fig. 2a. By its turn, the voltage level set to the reference electrode (RE_pH_) was derived from the DC supply (1.8 ​V) down converted to a level of 0.2 ​V by a voltage divider, followed by signal buffering by the same amplifier (AD8506) in order to impose a constant potential to the tested pH solutions, wherein the concentration of pH species (e.g., protons) produce a deviation from the neutral level over WE_pH_. After amplification, this deviation is digitized by a microcontroller (μC, ATtiny 20, Microchip, USA) at a rate of 16 samples *per* second (SPS) and resolution of 10-bit or, equivalently, a voltage resolution of 1.76 ​mV.

For lactate sensor readings, a similar circuit was used to set the reference potential (RE_LAC_) at 0.2 ​V, whereas a transimpedance amplifier (AD8506) with high DC gain (1000000x) was connected to WE_LAC_ to convert the ionic current present in solution due to lactate ionization into a voltage equivalent. This signal is then digitized by the microcontroller at a rate of 16 SPS and 10-bit resolution (or 1.75 ​nA in terms of current resolution). The μC is additionally responsible for collecting temperature measurements as it possesses an internal temperature indicator (chip die, range from −40 ​°C to +85 ​°C, resolution of 1 ​°C) working at rate of 1 SPS. All acquired samples are then sent to an NFC chip tag (NT3H1101, NXP, The Netherlands), becoming almost immediately accessible by the radiofrequency (RF) field generated with a smartphone. The same chip tag is also responsible for harvesting the RF energy and conversion to the DC supply level by connection to an external circular antenna made with enamelled wire (7 turns, 16 ​mm diameter). The minimum harvested voltage for device operation is 1.8 ​V, which multiplied by the total current consumption of the device (≈0.44 ​mA) translates into a power consumption of 0.79 ​mW. The full voltage harvesting profile obtained by this antenna configuration is further demonstrated in the *Results and discussion* section.

### 2.3 Circuit board design, TIAP embodiment, and smartphone's app

In terms of board fabrication technology, the electronic circuit was designed in Eagle (Autodesk, USA) as a double layered printed-circuit-board (PCB) with dimensions of 3.75 ​mm ​× ​6.5 ​mm and thickness of 1 ​mm. Copper traces with thickness of 150 ​μm were used for electrical signal routing with Ni/Au surface finishing over the exposed electronic pads. Liquid photoimageable soldermask was applied to both layers of the PCB to provide electrical protection and isolation, as well as facilitate automatic component assembly. Stainless-steel stencils were fabricated to allow the deposition of solder paste (HF 202, Multicore/Loctite, Germany) over the exposed pads only. Then the PCB was placed inside a pick-and-place machine (MC400, Manncorp, USA) for automatic component assembly instead of manual soldering due to the tiny packages for the selected chipsets (ball grid arrays). At the end, the PCB was placed inside a reflow oven (MC301, Manncorp) with peak temperature set at 300 ​°C to allow melting of the solder paste and fixation of the components to the respective pads with good solder connection.

Prior to PCB attachment to the TIAP structure, enamelled wires were manually soldered to the input pads dedicated to the chemical sensors (reference and working electrodes) with lead-free solder at a temperature of 330 ​°C. By its turn, the structure for the TIAP was 3D modelled in Solidworks (Dassault Systems, USA). The structure was printed using a fused deposition modelling 3D printer (Fortus 400 ​MC, Stratasys, USA) with a biocompatible material (ABS-M30i, ISO 10993 USP Class VI), whose Vicat softening temperature was 99 ​°C and tensile strength of 36 ​MPa. After printing, the structure was placed in a caustic soda (NaOH) solution to remove material debris, followed by wash and drying processes. The 3D printed structure was designed to facilitate housing of the NFC antenna on top and the PCB for the readout electronics in the open space located in the middle of the structure, as shown in Fig. 2b. Finally, the PCB was attached to the TIAP with the terminals of the antenna soldered to the respective pads and the distal end of each enamelled wire to the respective receptacle in the adapter for the electrochemical sensors located at the bottom of the TIAP structure (Fig. 2c). Polydimethylsiloxane material (PDMS, Sylgard 184) was additionally dropped over the entire structure to provide mechanical stability and biocompatibility with biological tissues. The final structure was cured in the same reflow oven for 30 ​min at 80 ​°C. Testing of the device and NFC interface was performed afterwards using a custom-designed smartphone's *app* developed in Android Studio (Google, USA) to receive the data packets sent from the assembled structure, as exhibited in Fig. 2d.

## 3 Results and discussion

**Fig. 3:**
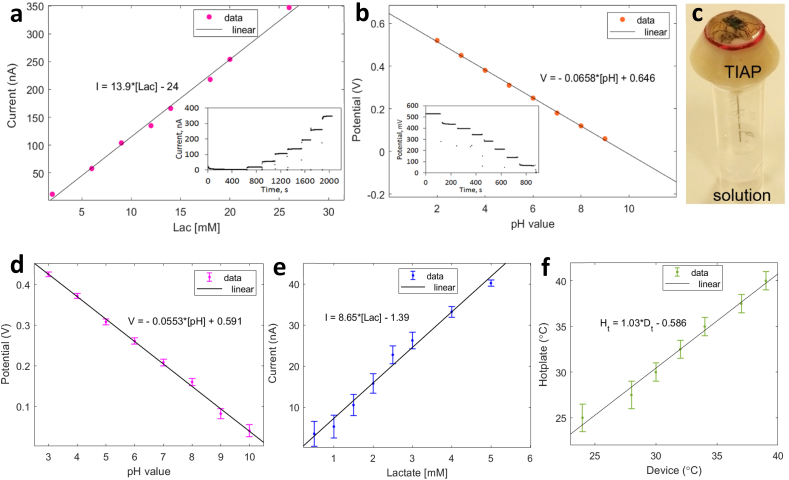
Illustration of: (a) lactate and (b) pH linear curve fit obtained with measurements collected by a potentiostat instrument (inset: time measurements for the different tested solutions); (c) TIAP device placed on top of a solution container with the needle-electrodes in contact with the liquid for NFC readings; (d) pH measurement curve and respective linear fit obtained by the NFC readings for solutions of pH 3, 4, 5, 6, 7, 8, 9 and 10; (e) lactate measurement curve and respective linear fit obtained by the device for solutions with concentration of 0.5, 1, 1.5, 2, 2.5, 3, 4 and 5 ​mM; (f) temperature calibration curve obtained by the TIAP when placed over an hotplate.

In the first set of laboratory experiments, the developed biosensors for lactate and pH were tested with prepared solutions in sequence and the respective response was detected by a commercial benchtop potentiostat (Ivium Technologies, The Netherlands). Fig. 3a and 3b shows the temporal recordings obtained by this process and the associated linear fit for lactate and pH respectively, when testing both analytes separately. For lactate, the working range tested was 26 ​mM, with the sensor exhibiting a good linear response between concentration levels and ionic currents detected, yielding a sensitivity of approximately 13.9 ​nA/mM. The deposition of an outer membrane for the lactate sensor was essential to provide both physical protection for the enclosed enzyme and biocompatibility with the surrounding body tissues. By its turn, the range of tested solutions for pH was between 2 and 9, obtaining again a good linear relationship between pH value and detected potential, producing a linear slope of 65.8 mV/unit pH. The fabricated IrOx films have proved to provide fast response times to pH solution changes (less than 1 ​s), as well as high sensitivity (that is, Nernstian response above 59 mV/unit pH) and minimal potential drift as observed by the stable staircase profile obtained for the different solution baselines.

After testing with the potentiostat, the biosensors were attached to the developed TIAP device and immersed on solutions contained inside Eppendorf tubes for contactless NFC readings, as shown in Fig. 3c. Each needle-like electrode was tested separately, with the pH one being tested first for solutions spanning unitary pH levels between 3 and 10. The linear profile detected is depicted in Fig. 3d as an average of the potential level recorded (and respective error bar) by the smartphone during 10 reading sequences separated by temporal windows of 10 ​s. The calculated sensitivity obtained was 55.3 mV/unit pH, which is lower than the sensitivity achieved by the potentiostat due to the limited electrical characteristics of the TIAP device in terms of harvested voltage level and signal acquisition performance. Nonetheless, the small error bars obtained in conjunction with the large potential difference between tested pH solutions both attest the functionality of the proposed device. Within this regard, the minimum discernible pH level that can be theoretically achieved by the TIAP device is 0.032, this by combining the sensitivity achieved during solution testing and the voltage resolution of the pH acquisition channel embedded inside TIAP's electronics.

For lactate testing, NFC readings were taken from solutions with lower concentration levels to reflect the physiological and pathological limits (namely: 0.5, 1, 1.5, 2, 2.5, 3, 4 and 5 ​mM), a process also repeated 10x thus yielding the profile exhibited in Fig. 3e. The achieved sensitivity was around 8.65 ​nA/mM, with error bars decreasing in magnitude towards higher concentrations of lactate. Again, the limitation of the embedded electronics can be accounted for this performance degradation relative to the potentiostat measurements, with minimum discrimination level obtained for lactate around 0.2 ​mM. By changing the resistance value controlling the amplification gain inside electronics, the dynamic range for the lactate readings can be adapted to the medical need at hands, resulting in different minimum discriminative and maximum measurable levels for lactate that are reported dissimilar between the physiological and pathological states. Temperature measurements were additionally performed by placing the device over a hotplate (model 442–0662, VWR, USA) and increasing the temperature from 25 to 40 ​°C in 1 ​°C steps (Fig. 3f). Slight temperature underestimates were detected by the TIAP device relative to the control levels (hotplate), with error bars set to levels of ±1.5 ​°C.

**Fig. 4:**
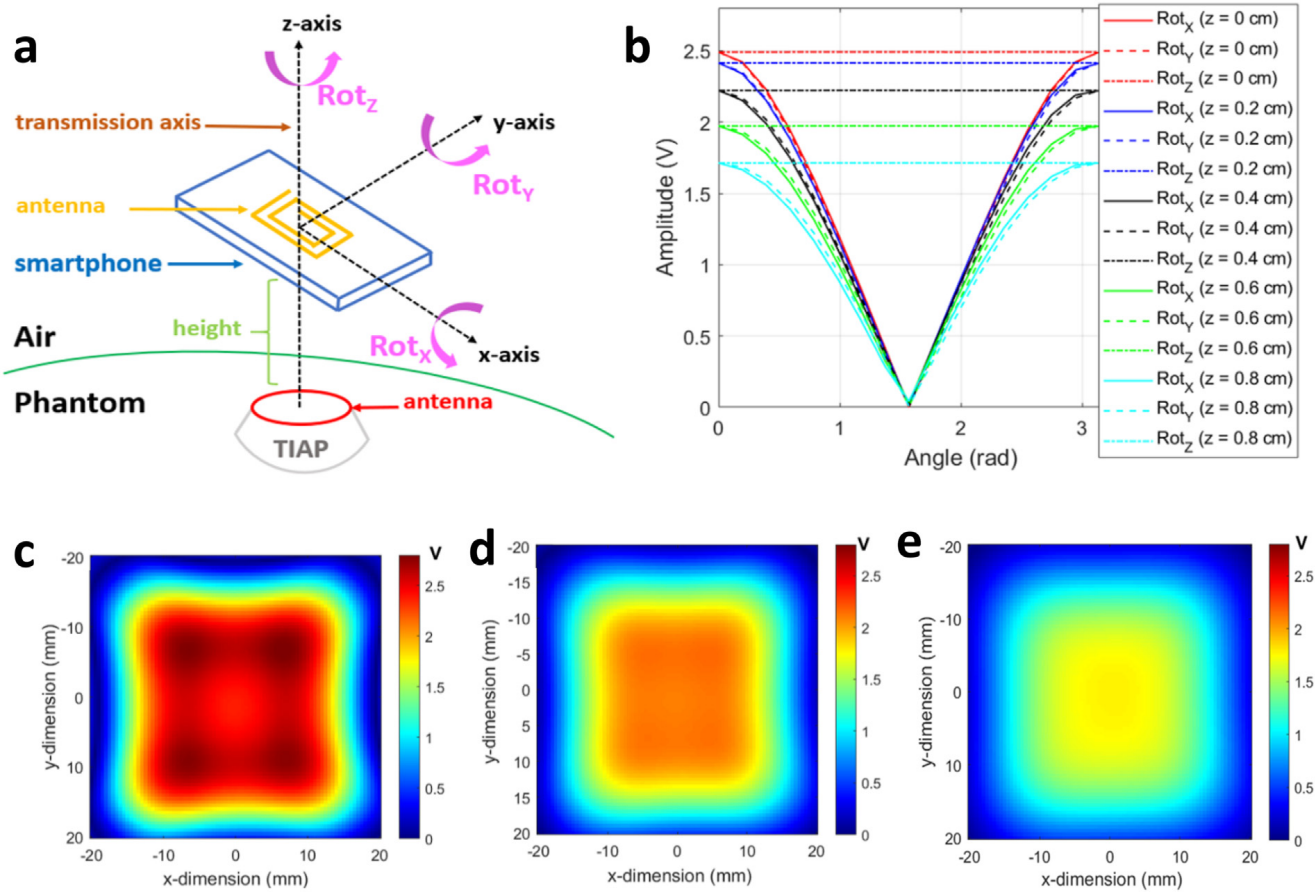
Illustration of: (a) schematic and referential axes for the calculation of the harvested voltage level by magnetic induction using the geometrical dimensions, number of turns and circulating current inside the smartphone's antenna in relation to the reception antenna on the TIAP; (b) voltage levels harvested by the TIAP at distinctive distances from the smartphone (in the z-axis), when both antennas are aligned through the transmission axis and subjected to different rotation angles (Rot_X_, Rot_Y_ and Rot_Z_) along the referential axes; (c) harvested voltage distribution map along the xy-plane at a distance TIAP - smartphone of 2.5 ​mm (height); (d) similar voltage distribution map obtained at a height of 5 ​mm; (e) voltage distribution map at a height of 7.5 ​mm.

Since previous measurements were performed with the smartphone in direct contact with the top surface of the TIAP structure (that is, ≈ zero-millimetre distance gap between these two entities), a further characterization was necessary to assess the harvested voltage profile at the TIAP as a function of the distance to the RF field source. Numerical simulations of the electromotive force generated by magnetic induction at the TIAP's antenna were performed in Matlab (Mathworks Inc., USA) following the mathematical model described in Ref. [22], this taking into consideration the geometries of the TIAP and smartphone's antennas in the 3D space spanned by Fig. 4a. Then, rotations of the smartphone's antenna relative to the TIAP in the three cartesian axis were simulated at a certain distance (height) between the geometric centres of the involved antennas when aligned along the transmission axis (also axes origin), thus obtaining the graph in Fig. 4b. Device functionality is assured for a maximum distance TIAP – smartphone of roughly 8 ​mm, as the harvested levels stay above 1.8 ​V. For the case of spatial misalignment between the antennas relative to the transmission axis (e.g., translation of the smartphone relative to the plane of the TIAP's antenna), Fig. 4c–e depict the two-dimensional distribution of the harvested voltage level obtained at three different heights (from 2.5 ​mm to 7.5 ​mm), with only the red and yellowish areas providing full harvesting capability to the TIAP from the RF field (≈10 ​mm^2^).

**Fig. 5:**
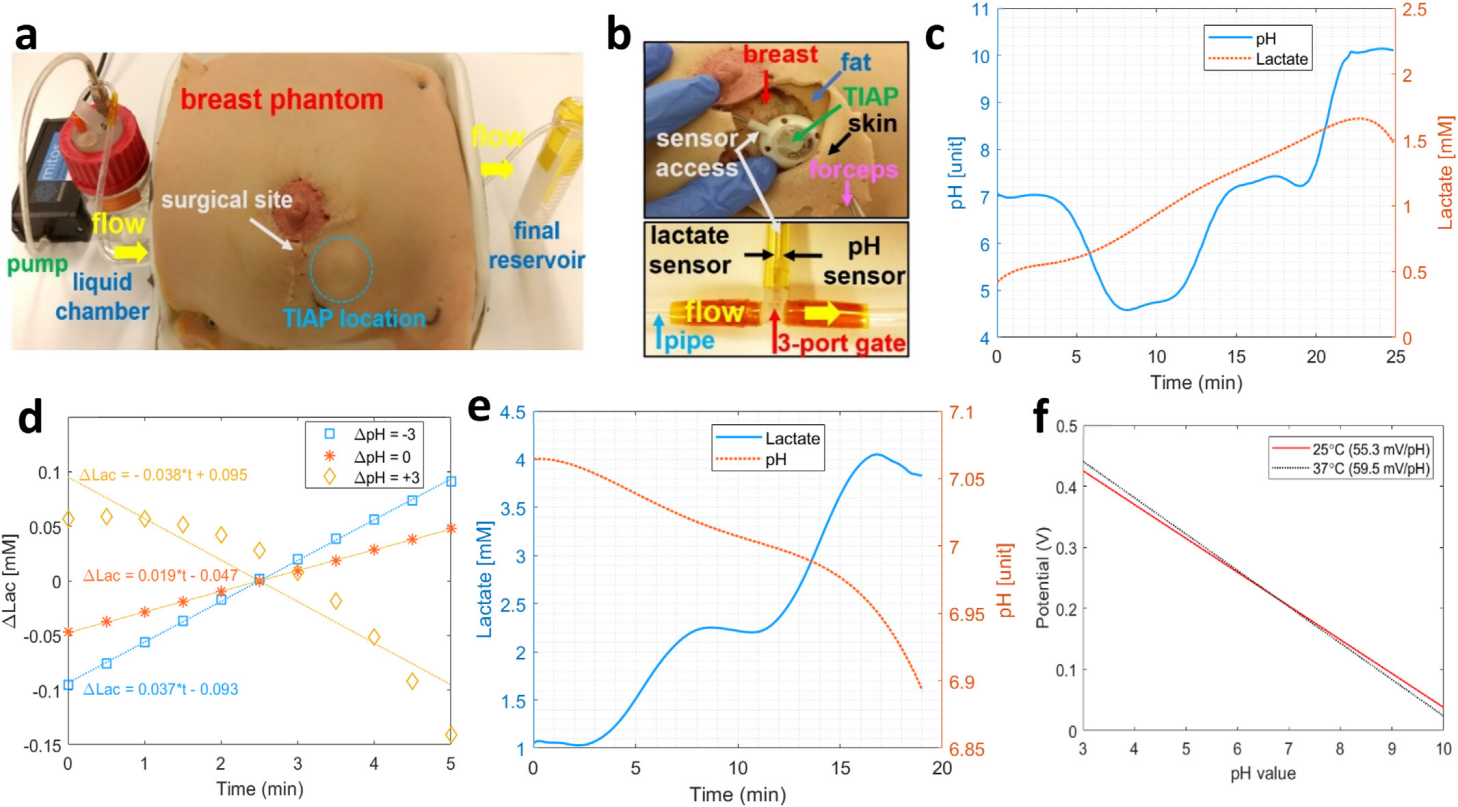
Illustration of: (a) breast phantom used for TIAP device testing, composed by a small electric pump that sets in motion the flow of liquid between the chamber and final reservoir internal to the phantom; (b) implantation of the TIAP device inside the breast phantom (5 ​mm deep, top image) and sensor access to the flow of circulating liquid through a 3-port gate (bottom); (c) extended temporal recording obtained by NFC readings for the variation of the pH level in circulation between neutral, acidic and basic phases (left axis), while recording the lactate level at the same time (right axis) for an imposed temperature of 37 ​°C; (d) variation of the lactate level detected by the TIAP between different pH injections (ΔpH); (e) temporal variation of the concentration of lactate in circulation obtained by NFC readings (left axis) between levels of 1 and 4 ​mM at temperature of 37 ​°C; (f) potential variation for pH obtained at two different temperatures (25 and 37 ​°C).

The next set of practical experiments involved the implantation of the TIAP device inside a realistic anatomical model of the human breast, with the needle-like electrodes in contact with an artificial flow of liquid solution set in motion by an external pump (Mitos Fluika Pump, Dolomite Microfluidics, UK), as depicted in Fig. 5a. The structure of the TIAP itself was placed subcutaneously under the artificial skin (gap distance ​≈ ​5 ​mm) during the surgical site intervention, whereas the sensors for pH and lactate were connected to the circulating flow underneath the phantom by a three-port gate (Fig. 5b) with imposed liquid temperature of 37 ​°C. Neutral pH was first injected in the liquid chamber and set into circulation, followed by the injection of solutions with pH 4, pH 7 (again) and pH 10, in roughly 5 ​min intervals, corresponding to the average time required to empty the liquid chamber. Fig. 5c shows the temporal evolution of the pH level detected by the TIAP during liquid circulation (left axis of the graph), whereas the right axis displays the variation of the lactate level, albeit not injected through the liquid chamber but still yielding the lower limit of detection by the TIAP (0.5–1.5 ​mM). In these circumstances, Fig. 5d depicts the variation of the detected lactate level by the device following each pH injection (with associated linear fit), revealing a higher slope for lactate in the transition from neutral to acidic pH (+37 ​μM/min, ΔpH ​= ​−3), followed by return to neutral pH (+19 ​μM/min, ΔpH ​= ​0) and inversion of the tendency in the transition from neutral pH to basicity (−38 ​μM/min, ΔpH ​= ​+3). Compensation for this deviation on the response of the lactate sensor can be achieved post-signal acquisition inside the *app* in order to allow accurate bi-modal sensing within the physiological range.

During sequential injection of lactate solutions (1, 2 and 4 ​mM) the solid line present on the graph of Fig. 5e is recorded, alongside with a subtle decrease in pH level (ΔpH ​≈ ​0.12) possibly due to the accumulation of lactic acid within the measurement setup, therefore post-signal compensation can be discarded for the pH channel during bi-modal sensing. At the end, and since the experimental tests were carried out with dissimilar temperature levels for the solutions (container: 25 ​°C, phantom: 37 ​°C), a comparison between the linear profiles detected for pH was performed and displayed in Fig. 5f, which reveals a drop in the pH sensitivity (4.2 mV/unit pH) for ΔT ​= ​12 ​°C. For lactate, the sensitivities between experiments remained fairly the same, therefore revealing the stability of the prepared lactate solutions (and biosensor) to cope with (small) temperature variations. Other performance metrics such as sensors’ durability and accuracy over time need to be further characterized inside a physiological medium to ascertain not only the biocompatibility property in the long term but also potential release of chemical compounds leading to interferences with other body analytes, molecules, and tissues or pO2 dependence. Since our measurements were not collected with *in vivo* phantoms, future work is required to assess properly the performance of the developed sensors using small animal models (e.g., rodents), as previously done by our research group with a slightly different implantable device [27]. To our best knowledge, studies on TIAP devices with incorporated electrochemical sensors are scarce or non-existent in the literature, which makes it difficult to compare the present device to some references on the field. Rather, these references traditionally use blood collection from the body through the TIAP structure (skin puncture) and analysis is performed off-body by standard microscopy techniques or other laboratory-intensive equipment. Even so, we present Table 1 as a comparison table for the current study, which contains mostly implanted devices that share similar transduction mechanisms, target analytes, powering interfaces or medical purposes that can be potentially adapted for an TIAP embodiment.

**Table 1:** Comparison table in terms of performance characteristics between the proposed device and others found in literature that are suitable for incorporation or adaptation inside an TIAP embodiment.

Ref.	Year	Physical device	Biomedical application	Transduction mechanism	Sensitivity/Performance	Power source	Distance device-reader	Target tissue
[35]	2012	Implantable batteryless capsule	Monitoring of impedance and pH (gastroesophageal reflux)	Voltammetry measurements	−51.7 ​mV/pH	Magnetic induction	<10 ​cm	Mannequin (*in vitro*) Pig model (*in vivo*)
[36]	2016	Implantable peripheral nerve cuff	Continuous monitoring of glucose (local inflammation)	Amperometry and electrochemical impedance spectroscopy	7.17 ​μA/mM.cm^−2^ LOD^a^ ​= ​10 ​μM	Tethered device (power cord)	–	Sciatic nerve of a rat (*ex vivo*)
[37]	2019	Needle-injectable sensing platform (sesame seed)	Closed-loop glucose control (diabetes)	Amperometry measurements	0.1045 ​nA/mg.dl^−1^ LOD ​= ​40 ​mg/dl	Radio-frequency (UHF^b^)	Under skin	Rat and swine models (*in vivo*)
[38]	2020	Micro-needle implantable sensor	Monitoring of partial pressure for oxygen – pO_2_ (metabolic disorders)	Cyclic voltammetry and chrono-amperometry	−2.496 ​nA/mmHg LOD ​= ​4.57 ​μM	Tethered device (power cord)	–	Quadriceps muscle of a rabbit (*in vivo*)
[39]	2020	Wien bridge oscillator circuit	Glucose and lactate monitoring (indwelling catheters)	Stimuli-responsive chemoresistors (molecular recognition)	33.9 mV_AC_/mM LOD ​= ​1.15 ​mM (glucose) 31.8 mV_DC_/mM LOD ​= ​0.54 ​mM (lactate)	Tethered device (power cord)	–	Liquid solutions
[40]	2020	Flexible and degradable polymeric substrate	Monitoring of nitric oxide (post-surgery health assessment)	Amperometry measurements	LOD ​= ​3.92 ​nmol	Battery	>1 ​m (transcutaneous connection to an off-body wireless module)	Cultured cells (*in vitro*) Joint cavity of rabbit (*in vivo*)
[41]	2021	Implantable optoelectronic catheter	Monitoring of tissue oxygenation (cardiopulmonary assessment post-surgery)	Light detection sensors (645 ​nm and 950 ​nm)	Penetration depth of 4–5 ​mm	Battery	>1 ​m (transcutaneous connection to an off-body wireless module)	Left ventricle of a rat (*in vivo*)
[33]	2021	Flexible and implantable device	Soft tissue pH and lactate measurements (cancer diagnosis)	Voltammetry and amperometry measurements	42 ​mV/pH (pH) 55.08 ​nA/mM (lactate)	Ultrasounds	50 ​mm	Breast phantom
[42]	2022	Implantable flow probe (biopsy needle)	Monitoring of microvascular blood flow (transplantation surgery)	Temperature-dependent resistance measurements	0.9–2.0 ​mm/s (flow velocity)	Battery	>1 ​m (transcutaneous connection to an off-body wireless module)	Porcine myo-cutaneous flap (*in vivo*)
This work	2022	Totally implanted access port device	pH and lactate measurements (interventional therapy)	Voltammetry and amperometry measurements	55.3 ​mV/pH LOD ​= ​0.032 (pH) 8.65 ​nA/mM LOD ​= ​0.2 ​mM (lactate)	Radio-frequency (NFC^c^)	8 ​mm	Perfused breast phantom

a LOD – Limit of detection.

b UHF – Ultra High Frequency.

c NFC – Near Field Communication.

Finally, regarding the transmission distance between the implanted TIAP and external smartphone accessing it (≈5 ​mm), this gap is still within the operability limits for wireless power and data telemetry delivery to the TIAP device, as well as for skin puncture to collect samples in some individuals. Depending on the thickness of the subcutaneous tissue layers composing the chest wall, the implantation depth for the TIAP can vary amongst patients due to the biological diversity factor. The depth of implantation reported in this study has been numerically and experimentally characterized based on the dimensions and number of turns composing the implanted antenna, which is located on the top of the TIAP structure. Higher harvested voltage levels can still be achieved (and consequently, deeper implantation distances inside the body) by increasing the number of turns for the antenna without modifying its overall planar dimension, at the expense of wrapping more enamelled wire along the lateral side of the 3D printed structure, thus creating a solenoid antenna whose electromotive forces originated by magnetic induction are proportional to the number and thickness of the composing wire loops [22].

## 4 Conclusion

A miniature, batteryless sensing device embodied into an TIAP structure has been demonstrated with power harvesting and data telemetry capabilities provided by the NFC interface of a smartphone. The performance of the proposed device compares favourably with a commercial potentiostat, though the small decrease in signal sensitivities for the tested biosensors is a direct consequence of employing ultra-low powered chipsets inside electronics. The developed pH and lactate biosensors were also electrochemically stable throughout the experiments and demonstrated good linear response for the relevant biological range under analysis. Therefore, on-demand pH and lactate readings can be obtained wirelessly from the proposed TIAP device without requiring skin puncture for sample collection, which may be used by clinicians to infer about metabolic processes occurring inside the human body, as well as enabling timely informed decisions about patient's health and administration of therapy in case of detected tumour aggressiveness or infection for internal body tissues.

## Funding

This work was supported by the 10.13039/501100000266Engineering and Physical Sciences Research Council [EP/L014149/1, EP/P012779] and the 10.13039/100010269Wellcome Trust Funding Programme [WTiTPA PSZ088] in the United Kingdom.

## Credit author statement

**Bruno Gil**: Conceptualization, Investigation, Data curation, Writing – original draft. **Benny Lo**: Software, Resources. **Guang-Zhong Yang**: Funding acquisition, Project administration, Writing – review & editing. **Salzitsa Anastasova**: Methodology, Validation, Visualization, Writing – original draft.

## Data availability

Data is available upon request from the corresponding authors.

## Declaration of competing interest

The authors declare that they have no known competing financial interests or personal relationships that could have appeared to influence the work reported in this paper.
